# Impact of introducing guidelines for thrombolysis of submassive pulmonary embolism at a large UK teaching hospital

**DOI:** 10.1186/cc14404

**Published:** 2015-03-16

**Authors:** GP Misselbrook

**Affiliations:** 1 University Hospitals Southampton NHS Foundation Trust, Southampton, UK

## Introduction

Pulmonary embolism (PE) is a significant cause of death with 10% of patients dying within 3 months [1]. Multiple studies now advocate the use of thrombolysis (TPA) in both massive and submassive PE [1,2]. This audit assessed the impact of introducing a guideline allowing for thrombolysis of submassive and massive PE at a large UK teaching hospital.

## Methods

**Figure 1:**
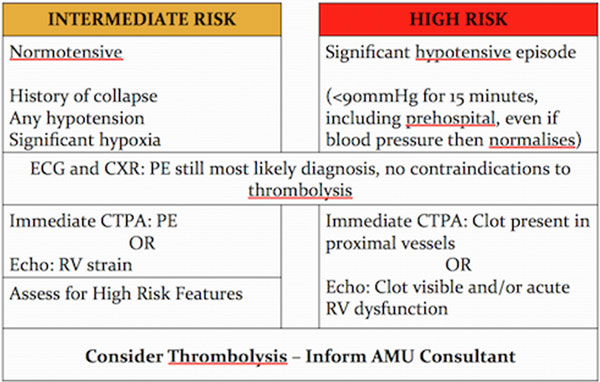
**New local guidelines**.

Retrospective data collection using notes and imaging to risk-stratify patients. First audit ran from January to June 2012. New guidance was introduced in March 2013 (Figure 1) after which a second cycle ran for a further 6 months.

## Results

Re-audit revealed 46 patients with radiological evidence of massive or submassive PE on CTPA (32% of all PEs). Ten patients had clinical features of submassive PE and nine presented as massive PE. Previous guidelines suggested consideration of TPA in only seven patients in 6 months. TPA was given to two patients; however, six patients had no contraindications to treatment (Table 1). Limitations to TPA administration were late recognition of submassive PE and inadequate knowledge of changes to guidelines.

**Table 1:** TPA decisions.

	Total	High risk	Intermediate risk
Considered TPA and given	2	2	0
Considered TPA but contraindications	1	1	0
Considered TPA and not given on balance	3	0	3
**Considered TPA and not given but fit criteria**	**3 **	**2 **	**1 **
Not considered TPA but contraindicated anyway	4	1	3
Not considered TPA and on balance would not be given	5	1	4
**Not considered TPA but fit criteria **	**1 **	**0 **	**1 **

## Conclusion

Delivering a service that offers TPA to patients with submassive PE significantly increases the need to consider this therapy. Introducing this service is only effective if doctors initially assessing these patients are aware of recent changes to guidelines for PE.

## References

[B1] Meyer G N Engl J Med 2014 370 1402 1411 10.1056/NEJMoa1302097 24716681

[B2] Kearon C Chest 2012 141 419 496S

